# Barnes on Diseases of Women

**Published:** 1874-05

**Authors:** 


*A Clinical History of the Medical and Surgical Diseases of Women.* By Robert Barnes, M. D., Lond., with one hundred and sixty-nine illustrations. Philadelphia: Henry C. Lea, 1874. Buffalo: Theo. Butler & Son.

Dr. Robert Barnes is well known for his writings upon Obstetric Surgery, and those who have read his former productions with pleasure and profit, will not in the least be disappointed in this, unless it is that in looking for a Clinical History of the Diseases of Women, they find a work which can almost approximate to the dignity of a Treatise.

As if conscious of his own authority upon the chosen subject, the author says what he has to say in a plain, straightforward manner, quoting but few authorities and relying upon no one for support. While he shows a more than ordinary intelligence upon American Medical writings, we are supprised to find no mention of Enucleation of Ovarian Tumors or of the eminent success which has attended the operations of Dr. Jas. P. White, of this city, in restoring chronic cases of inverted uteri.

The various subjects are upon the whole well considered, and this book is a monument of industrious, patient observation and study. Many of the illustrasions are new, and all are well selected. Dr. Barnes is doubtless one of the best living authorities upon Diseases of Women, and his book is one which will be consulted with pleasure by all in search of information upon the subject.

